# Optical Coherence Tomography Image Classification Using Hybrid Deep Learning and Ant Colony Optimization

**DOI:** 10.3390/s23156706

**Published:** 2023-07-26

**Authors:** Awais Khan, Kuntha Pin, Ahsan Aziz, Jung Woo Han, Yunyoung Nam

**Affiliations:** 1 Department of ICT Convergence, Soonchunhyang University, Asan 31538, Republic of Korea; awaiskhanfa14@gmail.com (A.K.); pin.kuntha145@gmail.com (K.P.); ahsanaziz37406@gmail.com (A.A.); 2 Department of Ophthalmology, Bucheon Hospital, Soonchunhyang University College of Medicine, Bucheon 14584, Republic of Korea; 3 Department of Computer Science and Engineering, Soonchunhyang University, Asan 31538, Republic of Korea

**Keywords:** optical coherence tomography, deep learning, convolutional neural network, feature selection, ant colony optimization, machine learning, age-related macular degeneration, branch retinal vein occlusion, central retinal vein occlusion, central serous chorioretinopathy, diabetic macular edema

## Abstract

Optical coherence tomography (OCT) is widely used to detect and classify retinal diseases. However, OCT-image-based manual detection by ophthalmologists is prone to errors and subjectivity. Thus, various automation methods have been proposed; however, improvements in detection accuracy are required. Particularly, automated techniques using deep learning on OCT images are being developed to detect various retinal disorders at an early stage. Here, we propose a deep learning-based automatic method for detecting and classifying retinal diseases using OCT images. The diseases include age-related macular degeneration, branch retinal vein occlusion, central retinal vein occlusion, central serous chorioretinopathy, and diabetic macular edema. The proposed method comprises four main steps: three pretrained models, DenseNet-201, InceptionV3, and ResNet-50, are first modified according to the nature of the dataset, after which the features are extracted via transfer learning. The extracted features are improved, and the best features are selected using ant colony optimization. Finally, the best features are passed to the k-nearest neighbors and support vector machine algorithms for final classification. The proposed method, evaluated using OCT retinal images collected from Soonchunhyang University Bucheon Hospital, demonstrates an accuracy of 99.1% with the incorporation of ACO. Without ACO, the accuracy achieved is 97.4%. Furthermore, the proposed method exhibits state-of-the-art performance and outperforms existing techniques in terms of accuracy.

## 1. Introduction

The human retina is essential for processing visual information. The inner surface of the eyeball is lined with a layer of photosensitive optical nerve tissues [1]. The retina receives the light focused by the lens, which produces neural signals. Inside the retina, the macula, responsible for sharp and sensitive vision, perceives visual details, colors, and light intensities. With the aid of the optical nervous system, the retina analyzes these data before transmitting neural impulses to the brain. Eye conditions, such as macular degeneration, which are often ignored, can cause total vision loss [2,3]. Ophthalmologists typically categorize these diseases into five classes. Age-related macular degeneration (ARMD), branch retinal vein occlusion (BRVO), central retinal vein occlusion (CRVO), central serous chorioretinopathy (CSCR), and diabetic macular edema (DME) are conditions that can lead to blindness [4]. These diseases affect the retina, the light-sensitive layer at the back of the eye, and can result in vision loss if left untreated [5].

Optical coherence tomography (OCT) is a noninvasive imaging technique widely used in ophthalmology for the diagnosis and monitoring of various eye diseases. It enables the generation of high-resolution 3D images that provide detailed insights into the structure and function of the human retina [6]. In particular, OCT has emerged as a gold standard for detecting and evaluating macular disorders such as diabetic macular edema (DME) and age-related macular degeneration (AMD), both of which can lead to vision impairment and blindness. By capturing precise images and allowing for early detection, OCT facilitates timely interventions, personalized treatment strategies, and improved patient outcomes [7,8]. Its remarkable capabilities have revolutionized the field of ophthalmology, enabling clinicians to deliver more accurate diagnoses and optimized care for individuals with retinal conditions [9].

However, the availability of supervised data in the medical field is limited, and these data require specialized knowledge. To overcome this challenge, various deep learning techniques have been developed [10]. One method involves increasing the number of training samples using data augmentation techniques, such as geometrical transformations of images or mimicking image distributions [11,12]. Another approach is unsupervised learning, which includes semi-supervised, multi-instance, and transfer learning (TL) [13]. TL has gained popularity in recent years because it effectively transfers model knowledge across different or unrelated tasks, requiring minimal retraining. Kermany et al. [14] demonstrated the effectiveness of TL in a study using deep learning models to classify normal eyes and eyes with three macular diseases, utilizing 4000 optical coherence tomography (OCT) images.

Over the last few decades, retinal OCT lesions have been detected using different approaches [15,16,17,18]. These diagnostic methods can be broadly categorized into two groups. The first group includes algorithms utilizing machine learning techniques to detect retinal OCT lesions. These methods employ image processing techniques such as local binary patterns, scale-invariant feature transformations, multiscale histograms of directed gradients, and other image processing techniques to extract image features.

To perform image classification tasks, several well-established machine learning (ML) techniques, such as support vector machines (SVM) and random forests, rely on feature extraction methods to generate discriminative representations. For instance, Alsaih et al. [19] developed a feature extraction pipeline that integrated the local binary mode of OCT and a directional gradient histogram to construct a distinctive feature set. This pipeline used a linear SVM classifier to predict the image categories. Similarly, Sun et al. [20] introduced a universal approach for retinal image alignment and cropping, followed by multiclass linear SVM classification to categorize dry ARMD and DME. Global image representation was obtained through sparse coding and a spatial pyramid.

Rong et al. [21] proposed an automated categorization approach for retinal eye diseases using a convolutional neural network (CNN)-based model. To reduce noise, the authors applied image denoising and used morphological dilation and thresholding to create a mask. For surrogate image generation, preprocessed images and masks were used to train the CNN model. The proposed model achieved an area under the curve (AUC) of 0.9856 and 0.9783 on the duke and local datasets, respectively. Karri et al. [17] effectively utilized a TL-based technology to classify three retinal eye diseases, namely normal studies, DME, and AMD. They used GoogleNet with TL and achieved a maximum accuracy of 96% by conducting ten experiments. Tan et al. [22] used a deep CNN model to detect AMD using OCT images. The authors utilized ten-fold validation and blindfold procedures to validate the model and reported accuracy indices of 95.45% and 91.17%, respectively. Gulshan et al. [23] proposed a deep learning approach for detecting DME and diabetic retinopathy from fundus images. The authors trained the model from scratch on raw input images, which required a large dataset and a longer training time. Using the EyePACS-1 dataset, they obtained a mean AUC of 0.991.

Lu et al. proposed a deep learning-based automated technique to detect different diseases using OCT images [24]. Their proposed model achieved a sensitivity and specificity of 94.4% and 97.3%, respectively, with an accuracy of 95.5% and AUC of 0.98. Alqudah et al. [25] demonstrated a CNN-based model for retinal illness diagnosis with a 97.10% accuracy index across five retinal eye diseases. Li et al. [26] proposed a TL technique based on VGG-16 for DME and AMD classification using OCT images. Their model achieved an accuracy of 98.6%, specificity of 99.4%, and sensitivity of 97.8%. Fang et al. [27] proposed an international fact checking network (IFCN) iterative fusion-based deep network for the automatic classification of four retinal eye diseases, achieving an accuracy of 93.25%. Rasti et al. [28] used an ensemble model of a CNN with a multiscale technique to achieve a classification accuracy of 99.39% for three classes of retinal eye diseases. Roy et al. [29] used a transfer learning-based technique to detect eye diseases and obtained a maximum accuracy of 93.32% for four classes using five deep learning models. Kermany et al. [3] proposed a deep learning-based approach for diagnosing four retinal disorders using the InceptionV3 pretrained CNN model with 96.60% accuracy.

Choudhary et al. [30] proposed a TL technique based on ResNet-50, InceptionV3, and VGG-19 for drusen, choroidal neovascularization, diabetic macular edema, and normal form classification using OCT images. The experiments were performed on a publicly available dataset consisting of 84,568 images, and the model VGG-19 achieved an accuracy of 99.17%. He et al. [31] demonstrated a Swin-Poly Transformer network method for classification of retinal OCT images. The experiments were conducted on OCT-C8 and OCT2017 with accuracy of 99.80% and 99.99% AUC. Karthik et al. [32] proposed an advanced diagnosis system for OCT image classification. They enhanced three ResNet architectures by replacing the residual connection with the EdgeEn block and cross-activation technique, resulting in improved contrast of the derivatives and sharper feature generation, ultimately leading to increased classification accuracy. Huang et al. [33] proposed GABNet, a novel lightweight classification model developed using the UCSD general retinal OCT dataset. The experiments were performed on 108,312 OCT images obtained from 4686 patients, with 3.7% improvement in classification accuracy.

The goal of this study is to overcome the limitations of existing approaches by proposing a new deep learning and ant colony optimization (ACO) framework for precise OCT image classification. The proposed framework includes the following steps:

- Modification of three pretrained models, InceptionV3, ResNet-50, and DenseNet-201, by adding a new layer that connects the preceding layers in terms of fully connected (FC) layers.
- The ACO method was used for feature selection. This method first involves the selection of features using ACO, which are then fine-tuned using an activation function.
- ACO was applied to deep learning models to compare their accuracy. Based on the accuracy, the best method was selected for the final classification.

## 2. Materials and Methods

### 2.1. Dataset

The OCT images used in this study were obtained from the Soonchunhyang University Bucheon Hospital and were labeled with eye diseases by ophthalmologists at the hospital. The images were collected and normalized with the approval of the Institutional Review Board (IRB). The dataset was collected twice, with the first collection comprising 2000 images in 2021, and the second collection comprising 998 images in 2022. The images were captured using a DRI-OCT (Topcon Medical System, Inc., Oakland, NJ, USA) camera, scanned across the range of 3–12 mm along both the horizontal and vertical directions. Moreover, the lateral resolution of the images was 20 µm, and in-depth resolution was 8 µm. The camera shooting speed was 100,000 A scans per second. The image resolutions varied in width from 1000 to 1050 pixels and in height from 300 to 350 pixels. This study was approved by the IRB of Soonchunhyang University Bucheon Hospital, Bucheon, Republic of Korea (approval number: 2021-05-001). All methods were performed in accordance with relevant guidelines and regulations.

### 2.2. Proposed Methodology

**Figure 1:**
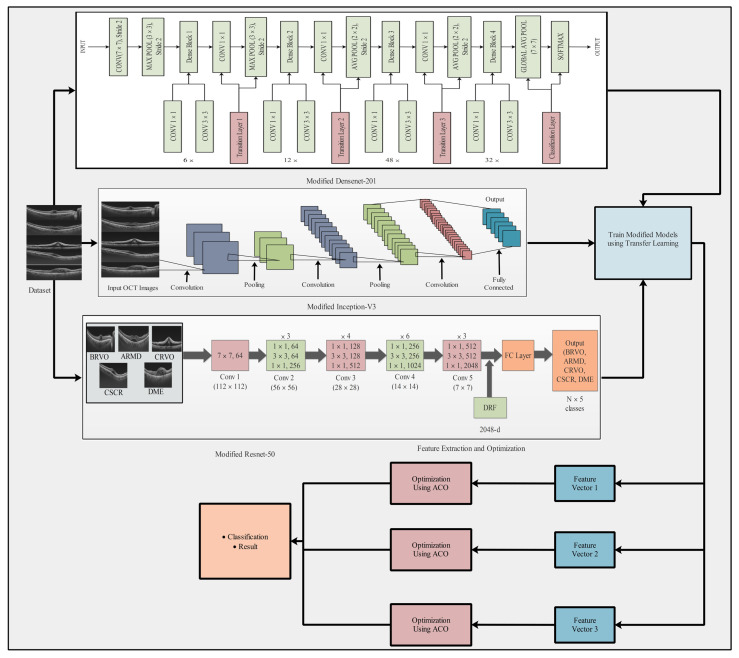
Flow diagram of the proposed method. Features are extracted by transfer learning, a feature vector is constructed, and ant colony optimization (ACO) is applied on the feature vector for optimization and final classification.

For OCT image classification, we propose a new deep learning method, which is represented in the main flow diagram in Figure 1. The methodology comprised several steps: data preprocessing, feature extraction employing pretrained models, feature optimization, and classification. This strategy implements advanced deep TL techniques with the objective of enhancing three pretrained models: ResNet-50, DenseNet-201, and InceptionV3 [34]. Upon extraction of the features from these modified models, the resulting vectors were refined using an advanced algorithm known as ACO. Finally, the refined features were subjected to multiclass classification to obtain the final results (Table 1).

### 2.3. Convolutional Neural Network

Deep learning is widely used in ML classification. One notable deep learning technique is the CNN [35,36]. This network uses a convolutional operator to extract features from image pixels, making it highly effective for image recognition, object detection, and classification. In addition, CNNs require minimal preprocessing compared to other classification algorithms. The network inputs are images, which are thereafter processed through various layers, such as convolutional, pooling, activation, and FC layers. The training and testing processes also involve multiple layers for image classification. In deep learning, various models have been proposed for classification, including ResNet, VGG, GoogleNet, and InceptionV3. In this study, we employed the three abovementioned pretrained models to perform classification tasks. The specifics of each model are described in the following sections.

### 2.4. Modified ResNet-50 Features

**Figure 2:**
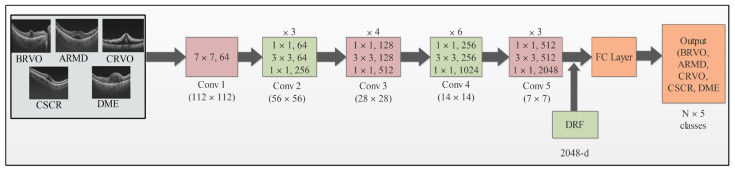
Modified proposed architecture of ResNet-50.

The ResNet architecture has been demonstrated to have superior performance by creating a more direct path for information flow throughout the network. This architecture also addresses the issue of the disappearing gradient in backpropagation. The utilization of shortcut connections, known as residual networks, allows the bypassing of layers, which may not be beneficial during training. The architecture of ResNet-50 includes a 7 × 7 convolution layer with 64 kernels, a stride 2, 3 × 3 max-pooling layer, a 7 × 7 avg-pooling layer with stride 7, 16 residual building blocks, and a final FC layer [37]. The ResNet-50 model has 23 million trainable parameters. In this study, we modified the pretrained ResNet-50 model by eliminating the final FC layer that initially supported 1000 object classes. Our focus was on an OCT image dataset comprising only five classes, and we incorporated a new FC layer with five layers. Subsequently, we applied deep TL techniques to train the modified model. Our transfer learning approach enabled us to generate a modified model suitable for feature extraction. This modified model was utilized to extract features from the global average pool layer, which resulted in feature vectors with dimensions of *N* × 2048. The architecture of the modified model is shown in Figure 2.

### 2.5. Modified DenseNet-201 Features

**Figure 3:**
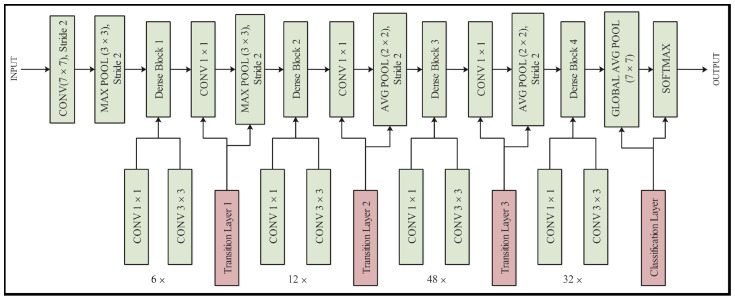
Modified architecture of DenseNet-201.

The DenseNet architecture is a deep neural network that utilizes a unique approach to connect layers, known as sequential concatenation. This method, which was first introduced in the ResNet model, improves traditional approaches by allowing the skipping of layers, resulting in a less complex and more efficient system. The network was originally trained on 1000 object classes and comprised 201 deep layers. Mathematically, this approach is defined as the concatenation of the output features from previous layers, instead of their summation. The DenseNet-201 architecture incorporates pooling blocks to downsample feature map sizes, resulting in reduced computational requirements. In addition, each layer within the DenseNet model has direct access to the original input image and gradients from the loss function. This feature results in significant gains in the computational efficiency. In this study, the DenseNet-201 architecture was modified for OCT image classification. The architecture of the modified model is illustrated in Figure 3. Initially, the model was trained on 1000 classes. After modifying the model, the FC layer was replaced with a new FC layer comprising only five classes. Training was performed using TL techniques, which involved the stochastic gradient descent (SGD) method, 100 epochs, a learning rate of 0.00001, and a minibatch size of 64. After training, the trained model was saved and utilized for feature extraction from the global average pooling layer. The feature vectors generated through this process were used for classification [38].

### 2.6. Modified InceptionV3

**Figure 4:**
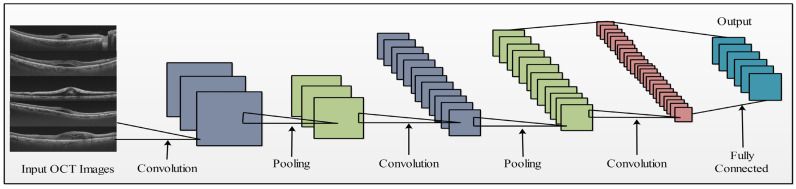
Modified proposed architecture of InceptionV3.

The architecture of this neural network was designed to handle image classification tasks; it was trained on 1000 object classes and comprised 48 layers. The input size of the images was 299×299×3. The network consists of several building blocks, such as convolutional layers, max-pooling, normal pooling, concatenation, dropouts, and FC layers [39]. The network began by passing the input image through three convolutional layers, each with a filter size of 3×3. Subsequently, the image was passed through the max-pooling layer with a window size of 3×3 and a stride of 2. The overall architecture of the network is mathematically represented by a combination of building blocks and their specific configurations. This model is an adapted version of a pretrained network used for OCT image classification tasks. In this study, an OCT image dataset was used to train the modified model. The input size for the modified model remains constant at 224×224×3 for the modified model. The modified model is shown in Figure 4, which comprises a convolution, max-pooling, average pooling, and new FC layer. TL techniques were applied to train the model using the features extracted from the average pooling layer. This process generates a feature vector with dimensions of N×1920.

### 2.7. Feature Optimization

Feature selection is a crucial aspect of pattern recognition, and various techniques have been used to optimize features, including particle swarm optimization, firefly algorithms, and genetic algorithms. In this paper, we propose ACO as a feature selection algorithm. The following sections explain the functioning of the ACO algorithm.

**Starting ant optimization:** The initial calculation of the number of ants is as follows:

(1) $$K_{n}=\sqrt{V\times d.}$$

The input feature vector is denoted by V, width of the feature vector is denoted by d, and number of random ants in the entire vector is denoted by $K_{n}$. Each feature in the feature vector represents one ant.

**Decision based on the probability:** The probability of an ant traversing from pixel $\left(e,f\right)$ to pixel $\left(g,h\right)$ is represented by $P_{ij}$. This probability can be calculated as follows:

(2) $$P_{ef}=\frac{(P_{ef}){\left(P_{ef}\right)}^{b}}{\sum {\left(P_{ef}\right)}^{b}({\left(v_{ef}\right)}^{b}u_{ef}{}^{\left(\Delta \right)}}$$

Here, the location of every feature is presented as ef ϵ Ω. The number of pheromones is represented by $P_{ef}$, $v_{ef}$ represents the visibility, and the value of $v_{ef}$ is defined by the following function:

(3) $$v_{ef}=P_{ef}$$

(4) Δ=0,π4,π2,3π4,π

**Rules of transition:** Mathematically, this rule is represented as follows:

(5) $$P=\left\{argu\left\{max_{j}\in S\left[\left(\rho_{ij}\right)b\left(u_{ij}\right)au_{ij}\left(\Delta \right)\right]\right\}\right\}$$

**Update pheromone:** During this phase, the ants are repositioned from their current coordinates $\left(i,j\right)$ to new coordinates to update the location of the features. Consequently, the pheromone trail is calculated and mathematically defined following each iteration.

(6) $$\delta_{ij}=\left(1-\mu \right).\delta_{ij}+\mu .\Delta \delta_{ij}$$

(7) $$\Delta \delta_{ij}=v_{ij}$$

During this process, the variable μ (0 < μ < 1) represents the pheromone loss proportion. The new pheromone value is determined after each iteration. Mathematically, this process can be represented as follows:

(8) $$\delta_{ij}=\left(1-\theta \right).\delta_{ij}+\theta .\delta_{0}$$

In this methodology, variable θ(0<θ<1)  denotes the degree of pheromone loss. The new pheromone values, as well as the starting pheromone values represented by $\delta_{0}$, were calculated for all features. Through this process, an optimal feature vector was obtained as the final output. The number of iterations in this study was set to 100. The vectors were selected after 100 iterations from the modified models, ResNet-50, InceptionV3, and DenseNet-201. During the analysis phase, certain redundant features in the selected vectors affected the recognition accuracy. Consequently, features in the range of 15–20% were removed. The remaining features that were determined to be the most beneficial through analysis were utilized for the final classification. The classification process involved the use of multiple classifiers, with the one yielding the highest accuracy selected as the most appropriate.

## 3. Results

### 3.1. Experimental Results and Analysis

The OCT image dataset used in this study was split into training and testing sets at a ratio of 80:20. The parameters for the training process included 100 epochs, 100 iterations, a minibatch size of 64, and a learning rate of 0.0001. A stochastic gradient descent SGD optimization algorithm employed SGD. A ten-fold cross-validation was performed, and multiple classifiers were evaluated using various metrics such as recall rate, precision, and accuracy. The simulation was performed using MATLAB 2022a. This study was performed on a Corei7 computer with 8 GB of RAM.

### 3.2. Results Proposed without ACO

**Figure 5:**
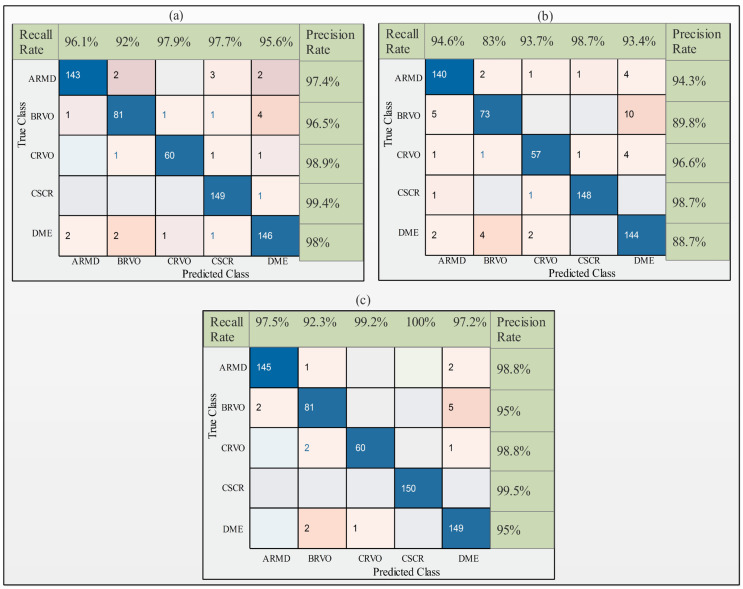
Confusion matrix of cubic support vector machine using deep learning models: (**a**) modified ResNet-50, (**b**) modified InceptionV3, and (**c**) modified DenseNet-201.

OCT images were employed in the experimental process, utilizing the modified deep learning models ResNet-50, InceptionV3, and DenseNet-201 to generate the outcomes without ACO. The performance of the ResNet-50 model is summarized in Table 2, which indicates that the highest accuracy achieved using ResNet-50 was 96.4% when employing the subspace k-nearest neighbor (KNN), with precision, recall, and AUC values of 96.3%, 96.2%, and 0.99, respectively. Cubic support vector machine (CSVM) attained the second best accuracy of 96.1%, accompanied by precision, recall, and AUC values of 96.2%, 95.8%, and 0.99, respectively. Figure 5a presents the confusion matrix for the ResNet-50 model.

The classification results of the InceptionV3 model without ACO are summarized in Table 3. The test features are passed to seven machine learning classifiers, and the results indicate that cubic SVM betters the other classifiers, achieving an accuracy of 93.5%. The precision, recall, and AUC were determined as 93.6%, 92.6%, and 0.99, and with computational time of 48.6 s. The subspace discriminant obtained the second best accuracy of 93.3%, accompanied by precision, recall, computational time, and AUC values of 93.6%, 92.6%, 156.1 s, and 0.99, respectively. The accuracy is better than the other classifiers but the computational time is high. Figure 5b displays the confusion matrices for the InceptionV3 model.

The results are summarized in Table 4, indicating that the modified DenseNet-201 model yielded the highest accuracy without ACO. CSVM achieved an accuracy of 97.4% using the test features, along with precision, recall, AUC, and computational time values of 97.4%, 97.2%, 1, and 46.1 s, respectively. The second best accuracy was achieved by quadratic QSVM of 97%, along with precision, recall, AUC, and computational time values of 97.02%, 96.7%, 1, and 42.1 s, respectively. Figure 5c shows the confusion matrix for the DenseNet-201 model without ACO.

### 3.3. Results Proposed with ACO

**Figure 6:**
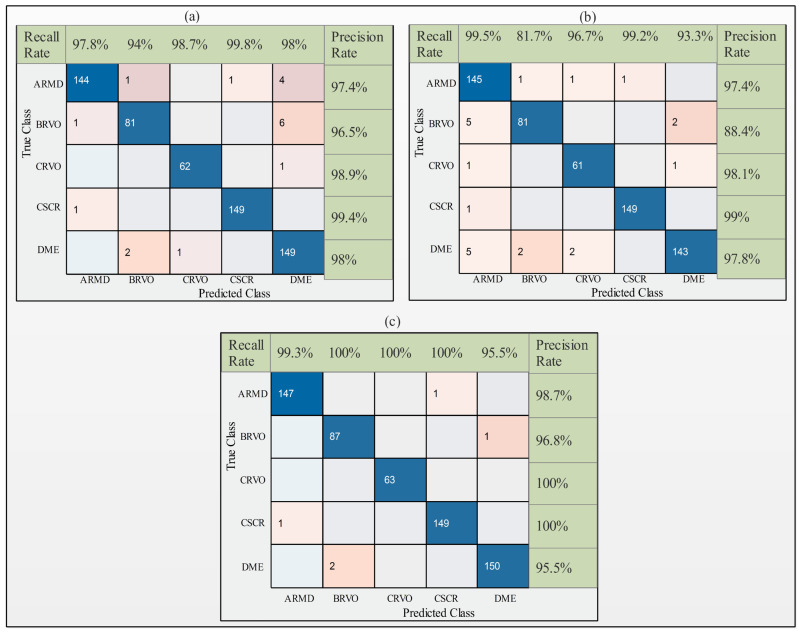
Confusion matrix of cubic support vector machine using deep learning models: (**a**) modified ResNet-50, (**b**) modified InceptionV3, and (**c**) modified DenseNet-201 with ACO.

Next, OCT images were utilized for the experimental process. The modified deep learning models ResNet-50, InceptionV3, and DenseNet-201 were employed to generate the results. The performance of the ResNet-50 model is summarized in Table 5. The results indicate that cubic support vector machine (CSVM) performed better than the other proposed ML classifiers. The highest accuracy obtained using ResNet-50 was 98% using cubic SVM. The precision rate, recall rate, and AUC were 98%, 97.6%, and 1, respectively. Quadratic SVM achieved the second best accuracy of 97.1%, with a precision rate, recall rate, and AUC of 98%, 97.6%, and 1, respectively. Linear SVM, cosine k-nearest neighbor (KNN), weighted KNN, subspace discriminant, and subspace KNN achieved accuracies of 95.4%, 94.3%, 92.4%, 75.1%, and 95.9%, respectively. The difference in accuracy between the quadratic and cubic SVM was approximately 0.4%. The confusion matrix for the ResNet-50 model and ACO is shown in Figure 6a.

Table 6 summarizes the classification results of the InceptionV3 model. The optimized features are passed to seven ML classifiers, and the results demonstrate that cubic SVM performs better than the other classifiers, obtaining an accuracy of 96.3%. The precision, recall, and AUC were 96.1%, 93.6%, and 1, respectively. The computational time noted was 48.6 s. Quadratic SVM achieved the second best accuracy of 95.9% with precision, recall, computational time, and AUC values of 97.1%, 96.1%, 64.9 s, and 1, respectively. The third best accuracy was 93.9%, achieved by linear SVM with a precision rate, recall rate, computational time, and AUC of 92.8%, 90.14%, 55.7 s, and 0.99, respectively. The confusion matrices for the InceptionV3 and ACO models are presented in Figure 6b.

**Figure 7:**
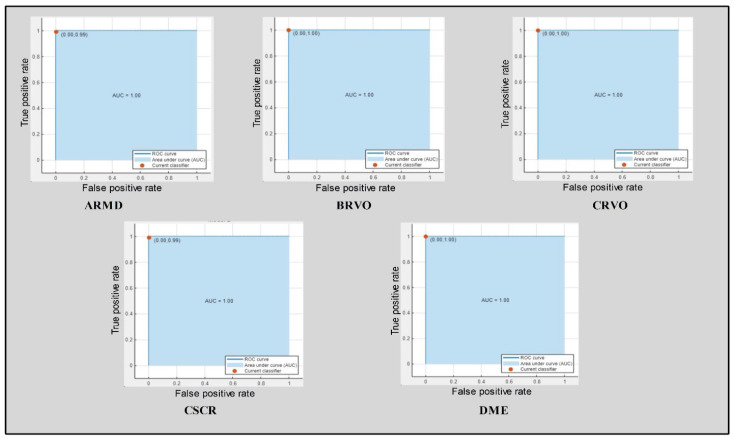
ROC plots for the selected OCT image classes of modified DenseNet-201 using cubic SVM after applying ACO.

**Figure 8:**
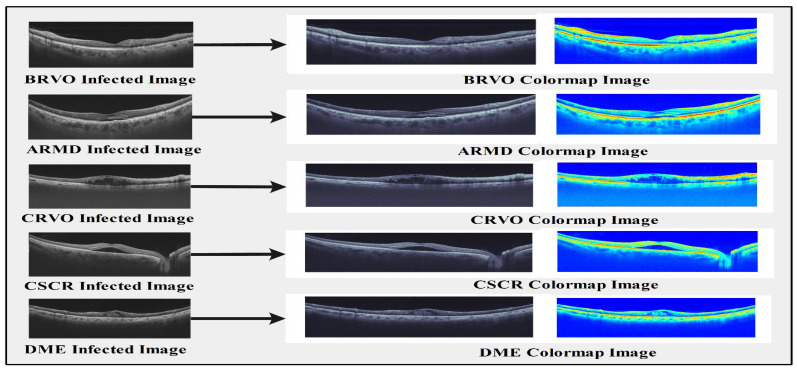
Sample of the OCT images from our proposed dataset and corresponding color images for understanding the input dataset images.

The results are summarized in Table 7. The best accuracy was achieved using the modified DenseNet-201 model and ACO. The best accuracy obtained by CSVM was 99.1% using the optimized features. The precision, recall, and AUC were 98.9%, 98.2%, and 1, respectively. The computational time noted was 47.1 s. The second best accuracy achieved by QSVM was 95.9%, with a precision rate, recall rate, computational time, and AUC of 98.5%, 97.7%, 66.3 s, and 1, respectively. The third best accuracy was 98.3%, achieved by the subspace discriminant with a precision rate, recall rate, computational time, and AUC of 98.22%, 98.06%, 60.2 s, and 1, respectively (Table 5, Table 6 and Table 7). The computational time was also considered for each classifier, and the optimal time was 50.4 s for CSVM. The confusion matrix for the DenseNet-201 model and ACO is shown in Figure 6c. The receiver operating characteristic curve (ROC) plots for the selected OCT image classes of the modified DenseNet-201 model using cubic SVM after applying ACO are shown in Figure 7. A sample of the OCT image dataset with the corresponding color images used to understand the input dataset images is shown in Figure 8.

## 4. Discussion

This section discusses the technical aspects and results of the proposed automated method for detecting retinal eye diseases using OCT images. The OCT dataset of retinal eye diseases with the five classes ARMD, BRVO, CRVO, CSCR, and DME was used in this study. A simple TL technique was employed for deep feature extraction, which was performed using modified ResNet-50, InceptionV3, and DenseNet-201 models with pretrained weights. The extracted feature vectors were thereafter passed to ACO for optimal feature selection. Subsequently, the selected feature vector was passed to seven machine learning algorithms for classification. The proposed automated methodology achieved an accuracy of 99.1%, which was the highest accuracy among all ML algorithms used. The best accuracy was achieved with cubic SVM using the DenseNet-201 model, with a computational time of 47.1 s. The cosine KNN classifier showed the worst accuracy when the DenseNet-201 model was applied, with an accuracy rate of 96.6% and a computational time of 60.7 s.

Notably, the modified DenseNet-201 model outperformed the other models, with ResNet-50 achieving an accuracy of 97.3%, and the InceptionV3 model achieving an accuracy of 96.32%. The worst performance was observed when using the subspace decrement classifier with the ResNet-50 model, with an accuracy of 75.1% and a computational time of 78.5 s. When using the InceptionV3 model, the best accuracy attained was 96.32% with a computational time of 48.6 s, whereas the subspace KNN classifier showed the worst accuracy of 89.8% and a computational time of 45.2 s. These results clearly demonstrate the effectiveness of the proposed feature selection and deep feature extraction techniques in the detection of retinal eye diseases. The results of the proposed method validated the effectiveness of the modified version of DenseNet-201, in which the softmax layer was replaced with a new one known as “new_softmax”. The softmax function is a common activation function used in the output layer of a neural network for multiclass classification problems and converts the network output into a probability distribution over the classes. A new softmax layer was added to change the classification task of the network for retinal eye disease detection.

The experimental results of the proposed methodology demonstrate its reliability for detecting retinal eye diseases, with the best multiclass classification accuracy of 99.1% with ACO, which is 2% better than the basic modified DenseNet-201. By automating the manual diagnosis process that requires experts, the proposed framework can facilitate timely and precise diagnosis of retinal eye diseases in hospitals. The proposed deep feature extraction and selection-based retinal eye disease detection and classification methods outperformed the most recent methods in the literature, as shown by the comparison in Table 8. This table shows that the proposed technique offers superior performance compared to the current techniques. Based on this discussion, it can be concluded that it is difficult to manually detect retinal eye diseases from OCT images, as it is a complicated and laborious task that can now be automated through the use of deep learning. Although various deep learning-based automated methods have been presented in the literature, the majority of them employ only deep CNN models and do not incorporate any feature selection techniques [40]. The proposed selection-based approach, which employs the modified ResNet-50, InceptionV3, and DenseNet-201 models, provides a reliable method for early and precise detection of various retinal diseases. In summary, the proposed automated method for detecting retinal diseases from OCT images offers a reliable and efficient solution for the timely and accurate diagnosis of such diseases (Table 8).

## 5. Conclusions

This study developed a novel method for detecting retinal diseases using retinal OCT images. This method was tested using a dataset of retinal OCT images in five classes: ARMD, BRVO, CRVO, CSCR, and DME. The proposed method comprised two main steps. In the first step, two deep features were extracted from retinal OCT images using modified ResNet-50, InceptionV3, and DenseNet-201 networks. In the second step, the ACO selection methodology was implemented to reduce redundant features. The selected high-level features were thereafter used to classify retinal eye diseases using different ML classifiers. The results of the proposed method showed that the combination of deep features and the proposed hybrid selection process achieved the best performance, with an average accuracy of 99.1%. A comparative study with existing approaches was also performed, and the results demonstrated that the proposed method outperformed other methods with the highest accuracy. Thus, it can be concluded that the proposed method is reliable and can accurately classify retinal eye diseases. In the future, this method can be further tested and evaluated on different datasets by including more eye diseases and different feature selection methods. The potential impact of this method is significant, as it can aid in the early and accurate diagnosis of retinal eye diseases, leading to prompt and effective treatment.

## Figures and Tables

**Table 1:** Distribution of OCT images with respect to classes.

Name of Classes	Total Number of Images	Test Images	Train Images
ARMD	738	148	590
BRVO	440	88	352
CRVO	313	63	250
CSCR	748	150	598
DME	759	152	607

**Table 2:** Proposed classification results of optical coherence tomography (OCT) images without ACO, using ten-fold cross-validation and ResNet-50.

Classifiers	Accuracy	Time	AUC	Precision Rate	Recall Rate
Cubic SVM	96.1%	32.7 s	0.99	96.24	95.88
Quadratic SVM	95.2%	31 s	0.99	95.4	94.98
Linear SVM	92.8%	29.3 s	0.99	93.2	92.1
Cosine KNN	92.1%	30.4 s	0.99	93	90.4
Weighted KNN	90.9%	29.3 s	0.99	92.2	89
Subspace KNN	96.4%	213.2 s	0.99	96.3	96.2
Subspace decrement	96%	159.5 s	0.99	95.8	95.6

**Table 3:** Proposed classification results of OCT images without ACO, using ten-fold cross-validation and InceptionV3.

Classifiers	Accuracy	Time	AUC	Precision Rate	Recall Rate
Cubic SVM	93.5%	44.5 s	0.99	93.6	92.6
Quadratic SVM	92.7%	33 s	0.99	92.9	91.5
Linear SVM	88.8%	29 s	0.98	89.4	87
Cosine KNN	86.6%	30.7 s	0.97	88.3	83.4
Weighted KNN	87%	30 s	0.98	88.3	84.3
Subspace KNN	87%	29.6 s	0.98	88.3	84.3
Subspace decrement	93.3%	156.5 s	0.99	93.2	92.8

**Table 4:** Proposed classification results of OCT images without ACO, using ten-fold cross-validation and DenseNet-201.

Classifiers	Accuracy	Time	AUC	Precision Rate	Recall Rate
Cubic SVM	97.4%	46.1 s	1	97.4	97.2
Quadratic SVM	97%	42.9 s	1	97	96.7
Linear SVM	94.6%	37 s	0.99	94.8	94.1
Cosine KNN	93.2%	32.1 s	0.99	94	91.7
Weighted KNN	94.6%	28.1 s	0.99	95.3	93.4
Subspace KNN	96.7%	183.5 s	0.99	96.9	96.1
Subspace decrement	96.4%	103.5 s	0.99	96.4	96.1

**Table 5:** Proposed classification results of optical coherence tomography (OCT) images using ten-fold cross-validation, ResNet-50, and ACO.

Classifiers	Accuracy	Time	AUC	Precision Rate	Recall Rate
Cubic SVM	97.3%	50.4 s	1	98	97.6
Quadratic SVM	97.6%	64.9 s	1	97.1	96.1
Linear SVM	95.4%	60.3 s	0.99	95	92.2
Cosine KNN	94.3%	61.4 s	0.99	94.8	91.4
Weighted KNN	92.4%	58.1 s	0.99	93.1	89.6
Subspace KNN	95.9%	68.5 s	0.99	94.9	94.4
Subspace decrement	75.1%	78.5 s	0.93	69.9	69.2

**Table 6:** Proposed classification results of OCT images using ten-fold cross-validation, InceptionV3, and ACO.

Classifiers	Accuracy	Time	AUC	Precision Rate	Recall Rate
Cubic SVM	96.3%	48.6 s	1	96.1	93.6
Quadratic SVM	95.9%	58.7 s	1	95.3	93.4
Linear SVM	93.9%	55.7 s	0.99	92.8	90.14
Cosine KNN	90.4%	60.2 s	0.99	90.3	86.2
Weighted KNN	89.9%	45.6 s	0.98	90.6	86.7
Subspace KNN	89.8%	45.2 s	0.97	89.8	88.3
Subspace decrement	93.3%	48.4 s	0.99	93.4	92.7

**Table 7:** Proposed classification results of OCT images using ten-fold cross-validation, DenseNet-201, and ACO.

Classifiers	Accuracy	Time	AUC	Precision Rate	Recall Rate
Cubic SVM	99.1%	47.1 s	1	98.9	98.2
Quadratic SVM	98.7%	66.3 s	1	98.5	97.7
Linear SVM	97.2%	59.8 s	1	97	95.1
Cosine KNN	96.6%	60.7 s	1	96.8	94.1
Weighted KNN	97.3%	59.1 s	1	97.7	96.3
Subspace KNN	98.2%	45.6 s	0.99	98.2	98.04
Subspace decrement	98.3%	60.2 s	1	98.22	98.06

**Table 8:** Comparison with existing methodologies.

Reference	Year	Method	Classes	No of Images	Accuracy (%)
N Rajagopalan et al. [41]	2020	CNN and deep learning	3	12,000	95.7%
Saraiva et al. [42]	2020	CNN	4	84,495	93.3%
DDJ Hwang et al. [43]	2021	Deep learning	2	3951	89.1%
Z Ai et al. [5]	2022	Transfer learning and CBAM	4	108,312	98.7%
J Han et al. [44]	2022	Transfer learning	4	4749	87.4%
**Proposed**	**-**	**Transfer learning and feature selection using ant colony optimization**	**5**	**2998**	**99.1%**

## Data Availability

The study’s data are available upon request from the corresponding author.
